# Safety Evaluation and Probiotic Potency Screening of Akkermansia muciniphila Strains Isolated from Human Feces and Breast Milk

**DOI:** 10.1128/spectrum.03361-22

**Published:** 2023-02-14

**Authors:** Fengyi Hou, Jingyi Tang, Yangyang Liu, Yafang Tan, Ye Wang, Lijun Zheng, Debao Liang, Yuqing Lin, Likun Wang, Zhiyuan Pan, Ruifu Yang, Yujing Bi, Fachao Zhi

**Affiliations:** a Guangdong Provincial Key Laboratory of Gastroenterology, Department of Gastroenterology, Institute of Gastroenterology of Guangdong Province, Nanfang Hospital, Southern Medical University, Guangzhou, China; b State Key Laboratory of Pathogen and Biosecurity, Beijing Institute of Microbiology and Epidemiology, Beijing, China; c Guangzhou ZhiYi Biotechnology Co., Ltd., Guangzhou, China; Chengdu University

**Keywords:** *Akkermansia muciniphila*, safety evaluation, breast milk, genetic stability, drug resistance, probiotics

## Abstract

Akkermansia muciniphila is considered a next-generation probiotic because of its immense potential to regulate disorders. We isolated 31 strains of *A. muciniphila* from feces or breast milk of healthy people. After genome sequencing, assembly, and analysis, we selected six strains (AM01 to AM06) for further exploration. We first analyzed their general characteristics, including morphological description, growth characteristics, and physiological and biochemical characteristics, and then confirmed their genetic characteristics, including GC content, putative virulence factors, and antibiotic resistance genes. We next investigated the tolerance of these strains to artificial gastric and intestinal fluids and bile salts to evaluate their survival potential in the digestive tract. Drug sensitivity tests were also conducted based on the analysis of the antibiotic resistance genes of these strains. Furthermore, we examined the genetic stability and acute toxicity of two strains (AM02 and AM06) in mice. Finally, the safety of AM06 was evaluated in normal mice and nude mice. AM06 exhibited adaptability to pH changes. Since AM02 and AM03 showed more resistance to antibiotics than AM01 and AM04 to AM06, their potential clinical application may be limited. Both AM02 and AM06 were genetically and phenotypically stable and safe in normal mice, and AM06 was safe in nude mice. Considering all this together, AM06 is a safe *A. muciniphila* strain and exhibits a great potential for use as a probiotic strain among the isolated strains.

**IMPORTANCE** In this study, we isolated 30 strains of Akkermansia muciniphila from different samples of human feces, and for the first time we isolated an *A. muciniphila* strain from human breast milk. This isolation verified the existence of microbes in human breast milk, which suggests that *A. muciniphila* can be vertically propagated from mother to infant and participates in the formation of the early gut microbiome. We then systematically evaluated the potential for use as a probiotic of this *A. muciniphila* strains according to the FAO/WHO recommendation. We confirmed that the AM06 strain isolated from breast milk has no virulence factors and is genetically stable and nonpathogenic for both normal mice and nude mice. Moreover, its tolerance to pH changes and bile salts indicates its desirable probiotic properties. Thus, we propose that the AM06 strain of *A. muciniphila* is safe for use as a probiotic candidate.

## INTRODUCTION

As a next-generation probiotic candidate, Akkermansia muciniphila has attracted widespread attention since its isolation from a human fecal sample for the first time in 2004 (1). It was a unique species in the family *Akkermansiaceae* until the isolation of Akkermansia glycaniphila from python feces in 2016 (2). A study using MUC-1437 as a 16S rRNA target probe of *A. muciniphila* reported that >1% of the total fecal cells were *A. muciniphila* (3). *Akkermansia* colonizes the human intestinal tract, increasing to the highest abundance level in the first year (4). Studies have shown that *A. muciniphila* can be detected in human breast milk, and this is supported by the fact that *A. muciniphila* can metabolize human milk oligosaccharides (5, 6). All these results suggest that *A. muciniphila* propagates vertically from breast milk to the intestinal tract and participates in forming the early gut microbiota.

By colonizing the human gastrointestinal tract, *A. muciniphila* specializes in mucin degradation, which may be attributed to a panel of enzymes (7). Nevertheless, *A. muciniphila* also promotes mucus production based on regulation of the differentiation of gut epithelial cells, influencing the gut barrier integrity and mucosal homeostasis (8). As an important role in gut microbiota, fluctuation of *A. muciniphila* abundance may be involved in metabolic disorders, immune diseases, and cancers. It has been reported that oral intake of living or pasteurized *A. muciniphila* regulates host metabolism by lowering serum triglyceride levels and enhancing insulin sensitivity, leading to a reduction in body mass gain in mice (9). Administration of *A. muciniphila*-derived extracellular vesicles was found to improve glucose tolerance and reduce body mass gain in mice with high-fat-diet-induced diabetes (10). However, some studies have contradicted the role of *A. muciniphila* in colitis and inflammatory bowel disease (IBD), which may be attributed to strain differences (11, 12). We also observed enrichment of *A. muciniphila* in patients who responded to immune checkpoint inhibitors (13, 14). Administration of *A. muciniphila* could improve the antitumor efficacy of PD-1 blockade (14). All these data indicate *A. muciniphila* as a potential probiotic.

In this study, we isolated 30 colonies (designated AM01 to AM05 and AM07 to AM31) of *A. muciniphila* from healthy human feces and for the first time isolated an *A. muciniphila* strain (designated AM06) from healthy human breast milk. We evaluated their potential for use as a probiotic. At the strain level, we systematically evaluated the *A. muciniphila* strains, including their general characteristics, genetic stability, antibiotic resistance, tolerance to artificial gastrointestinal fluid, and animal toxicity. Finally, we screened the AM06 strain isolated from breast milk as a candidate strain for further investigation.

## RESULTS

### Genetic characteristics of *A. muciniphila* strains.

**FIG 1:**
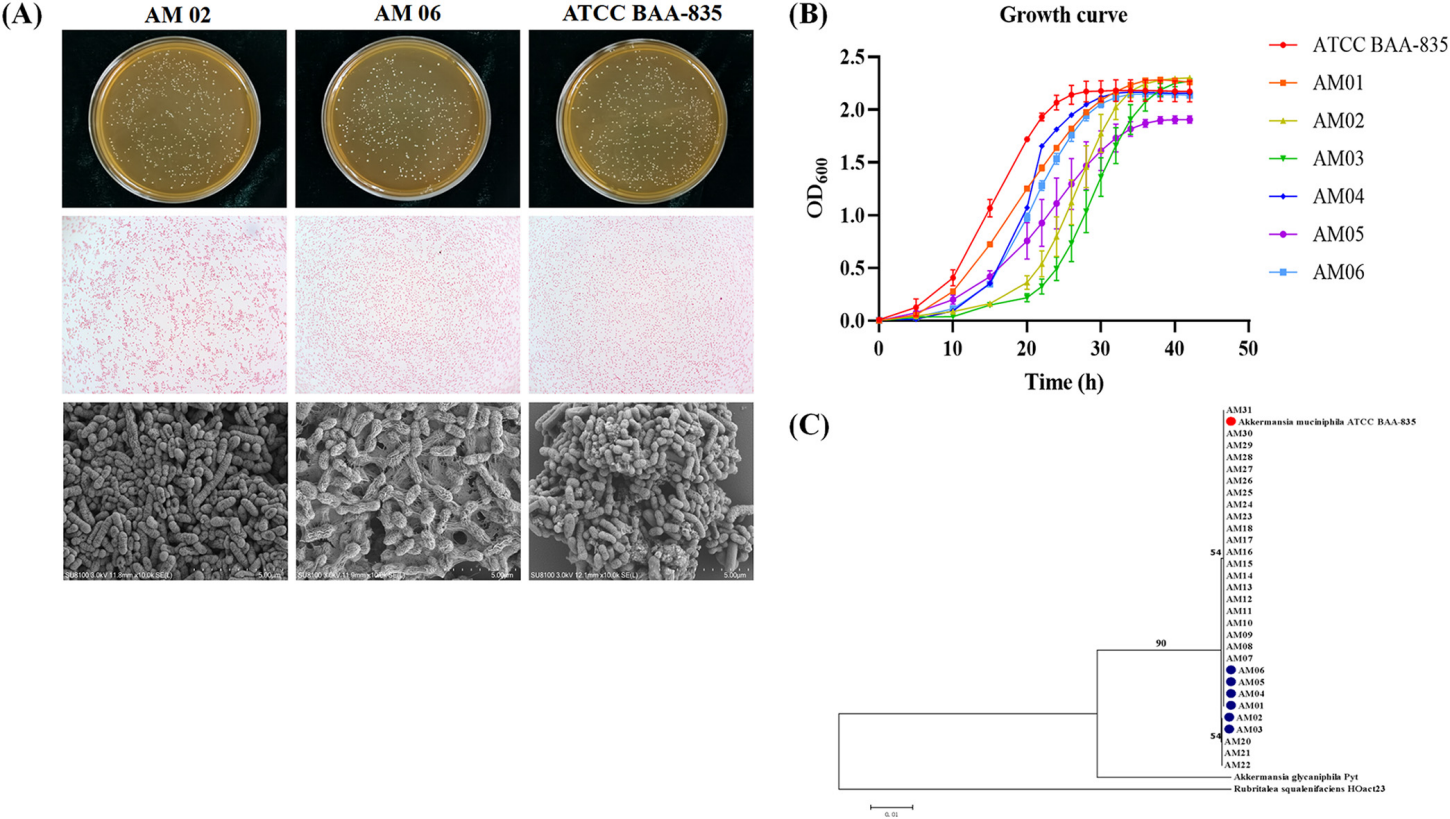
General characteristics of *A. muciniphila* strains. (A) Bacterial colonies on agar medium, Gram staining under an optical microscope, and morphological characteristics of bacterial cells under a scanning electron microscope of *A. muciniphila* AM02 and AM06 were identical to those of *A. muciniphila* ATCC BAA-835. (B) Growth curves of *A. muciniphila* AM01 to AM06 and strain ATCC BAA-835. (C) Phylogenetic tree of *A. muciniphila* AM01 to AM31 and strain ATCC BAA-835.

In total, 31 *A. muciniphila* strains (designated AM01 to AM31) were isolated in this experiment, and the sample origins are listed in Table 1 and Table S1 in the supplemental material. We aligned the 16S rRNA gene sequences of 31 isolated *A. muciniphila* colonies, *A. muciniphila* type strain ATCC BAA-835, *A, glycaniphila* strain Pyt, and Rubritalea squalenifaciens strain HOact23. A phylogenetic tree was generated using the neighbor-joining method by use of the MEGA 7 software (Fig. 1C). Strains AM01 to AM31 and ATCC BAA-835 were identified as the same species of *A. muciniphila*. AM02, AM03, and AM19 to AM22 were on the same branch, whereas AM01, AM04 to AM18, AM23 to AM31, and ATCC BAA-835 were on a different branch (Fig. 1C). Some strains were isolated from the same feces sample; for example, AM01 and AM07 were from volunteer 1 (Table S1). Therefore, we selected AM01 to AM06, which were isolated from six different samples, for further exploration.

**TABLE 1:** Sample origin and homology of strains AM01 to AM06 and ATCC BAA-835^*a*^

Parameter	Result for indicated strain						
	ATCC BAA-835	AM01	AM02	AM03	AM04	AM05	AM06
Sample origin	ATCC	Feces (26, M)	Feces (21, M)	Feces (30, M)	Feces (33, F)	Feces (29, M)	Breast milk (32, F)
Chromosome length (bp)	2,639,830	2,640,359	2,826,486	2,825,705	2,642,137	2,641,434	2,640,261
GC content (%)	55.73	55.75	55.31	55.31	55.73	55.73	55.73
ANI (%) between strains and ATCC BAA-835	100	99.99	97.31	97.30	99.97	99.98	99.99

a “Feces (26, M)” means that the strain indicated was isolated from the feces of a 26-year-old male human, and “Breast milk (32, F)” means that AM06 was isolated from the breast milk of a 32-year-old female human. Other abbreviations in the same row are synonymous.

The complete genomes of AM01 to AM06 and ATCC BAA-835 were contained in single chromosomes, and their lengths were 2,640,359, 2,826,486, 2,825,705, 2,642,137, 2,641,434, 2,640,261, and 2,639,830 bp, respectively (Table 1). The average GC content of AM01 to AM06 ranged from 55.31% to 55.75%, which was consistent with that of ATCC BAA-835 (55.73%) (Table 1). The average nucleotide identity (ANI) for AM01 to AM06 and ATCC BAA-835 ranged from 97.30% to 99.99% (Table 1).

There were 22 putative virulence genes in the genomes of AM01, AM04, AM05, and AM06 and 23 in those of AM02 and AM03 based on a minimum of 50% homology with genes in the Virulence Factors of Pathogenic Bacteria (VFDB) (Table S2). Most of these putative virulence genes were involved in the formation of cellular structures and in physiological activities. None of these putative virulence factors had been reported to play a role in the pathogenicity of *A. muciniphila*, which indicates that all the AM01 to AM06 strains were nontoxigenic at the genetic level.

### Morphological description of *A. muciniphila* AM01 to AM06.

All the AM01 to AM06 strains were obligate anaerobes and showed good growth on basal mucin medium and brain heart infusion (BHI) medium at 37°C. They were nonmotile, circular, protuberant, smooth, and opaque on the BHI agar medium containing 4.4 g/L *N*-acetylglucosamine and 6 g/L threonine (Fig. 1A; Fig. S1). The bacterial cells were Gram negative, oval shaped, and covered by filaments, as observed under an optical microscope and a scanning electron microscope (Fig. 1A; Fig. S1). The morphology of strains AM01 to AM06 was consistent with that of strain ATCC BAA-835 (1).

### General characteristics of AM01 to AM06.

AM01 to AM06 and ATCC BAA-835 grew well on culture medium containing no animal-derived components. The growth rates were slightly different among the strains. ATCC BAA-835, AM01, AM04, and AM06 reached the logarithmic phase within a short time period (Fig. 1B). However, all strains reached a growth plateau around an optical density at 600 nm (OD_600_) of 2.0 (Fig. 1B).

Carbon source utilization analyses indicated that AM01 to AM06 and ATCC BAA-835 differed in their ability to utilize β-cyclodextrin, gentiobiose, d-glucosaminic acid, glycerol, maltose, α-methyl-d-galactoside, β-methyl-d-galactoside, isomaltulose, l-rhamnose, turanose, glyoxylic acid, α-hydroxybutyric acid, l-malic acid, asparagine, and phenylalanine (Table 2).

**TABLE 2:** Substrates oxidized by *A. muciniphila* AM01 to AM06 and ATCC BAA-835 in Biolog AN MicroPlate test panel^*a*^

No.	Substrate	Substrate oxidized by indicated strain^*b*^						
		ATCC BAA-835	AM01	AM02	AM03	AM04	AM05	AM06
1	ddH_2_O	−	−	−	−	−	−	−
2	Acetyl galactosamine	+	+	+	+	+	+	+
3	*N*-Acetyl-d-glucosamine	+	+	+	+	+	+	+
4	Mannosamine	+	+	+	+	+	+	+
5	Ribitol	−	−	−	−	−	−	−
6	Amygdalin	−	−	−	−	−	−	−
7	d-Arabitol	−	−	−	−	−	−	−
8	Arbutin	−	−	−	−	−	−	−
9	Cellobiose	+	+	+	+	+	+	+
10	α-Cyclodextrin	−	−	−	−	−	−	−
11	β-Cyclodextrin	−	−	−	−	/	−	−
12	Dextrin	+	+	+	+	+	+	+
13	Hexanehexol	−	−	−	−	−	−	−
14	I-Erythritol	−	−	−	−	−	−	−
15	Fructose	+	+	+	+	+	+	+
16	Fucose	+	+	+	+	+	+	+
17	d-Galactose	+	+	+	+	+	+	+
18	d-Galacturonic acid	+	+	+	+	+	+	+
19	Gentiobiose	−	−	−	−	/	−	−
20	d-Gluconic acid	−	−	−	−	−	−	−
21	d-Glucosaminic acid	/	−	/	/	/	−	−
22	α-d-Glucose	+	+	+	+	+	+	+
23	Glucose-1-phosphate	−	−	−	−	−	−	−
24	Glucose-6-phosphate	−	−	−	−	−	−	−
25	Glycerol	−	−	−	−	/	−	−
26	d,l-α-Glycerophosphate	−	−	−	−	−	−	−
27	m-Myoinositol	−	−	−	−	−	−	−
28	α-d-Lactose	+	+	+	+	+	+	+
29	Lactulose	+	+	+	+	+	+	+
30	Maltose	+	+	+	/	/	/	+
31	Maltotriose	+	+	+	+	+	+	+
32	d-Mannitol	−	−	−	−	−	−	−
33	d-Mannose	+	+	+	+	+	+	+
34	d-Melezitose	−	−	−	−	−	−	−
35	d-Melibiose	−	−	−	−	−	−	−
36	3-Methyl-d-glucose	+	+	+	+	+	+	+
37	α-Methyl-d-galactoside	−	−	−	−	−	/	−
38	β-Methyl-d-galactoside	+	/	/	/	/	+	+
39	α-Methyl-d-glucoside	−	−	−	−	−	−	−
40	β-Methyl-d-glucoside	−	−	−	−	−	−	−
41	Isomaltulose	+	/	/	/	+	+	+
42	d-Raffinose	−	−	−	−	−	−	−
43	l-Rhamnose	/	/	/	/	/	−	/
44	Salicin	−	−	−	−	−	−	−
45	d-Sorbitol	−	−	−	−	−	−	−
46	Stachyose	−	−	−	−	−	−	−
47	Sucrose	−	−	−	−	−	−	−
48	d-Trehalose	−	−	−	−	−	−	−
49	Turanose	+	+	+	+	/	+	+
50	Ethanoic acid	−	−	−	−	−	−	−
51	Formic acid	−	−	−	−	−	−	−
52	Fumaric acid	−	−	−	−	−	−	−
53	Glyoxylic acid	/	−	−	−	/	/	−
54	α-Hydroxybutyric acid	/	−	−	−	−	−	−
55	β-Hydroxybutyric acid	−	−	−	−	−	−	−
56	Itaconic acid	−	−	−	−	−	−	−
57	α-Ketobutyric acid	/	/	/	/	/	/	/
58	α-Oxopentanoic acid	/	/	/	/	/	/	/
59	d,l-Lactic acid	−	−	−	−	−	−	−
60	l-Lactic acid	−	−	−	−	−	−	−
61	d-Lactate methyl ester	−	−	−	−	−	−	−
62	d-Malic acid	−	−	−	−	−	−	−
63	l-Malic acid	+	+	+	+	+	/	+
64	Propanoic acid	−	−	−	−	−	−	−
65	Pyruvic acid	/	/	/	/	/	/	/
66	Pyruvic acid methyl ester	−	−	−	−	−	−	−
67	d-Saccharic acid	−	−	−	−	−	−	−
68	Succinamic acid	+	+	+	+	+	+	+
69	Glycerol succinate	+	+	+	+	+	+	+
70	Monomethyl succinate	+	+	+	+	+	+	+
71	m-Tartaric Acid	−	−	−	−	−	−	−
72	Glycuronic acid	−	−	−	−	−	−	−
73	l-Ammonium propionate	−	−	−	−	−	−	−
74	Alanine	−	−	−	−	−	−	−
75	l-Glyceryl l-glutamine	−	−	−	−	−	−	−
76	l-Propylamine l-histidine	−	−	−	−	−	−	−
77	l-Propylamine l-threonine	−	−	−	−	−	−	−
78	Asparagine	−	−	−	−	/	−	−
79	Glutamic acid	−	−	−	−	−	−	−
80	l-Glutamine	−	−	−	−	−	−	−
81	Glycinyl-l-aspartic acid	−	−	−	−	−	−	−
82	Glycinyl-l-glutamine	−	−	−	−	−	−	−
83	Glycinyl-l-methionine	−	−	−	−	−	−	−
84	Glycinyl-l-proline	−	−	−	−	−	−	−
85	Methionine	−	−	−	−	−	−	−
86	Phenylalanine	−	−	−	−	/	−	−
87	Serine	−	−	−	−	−	−	−
88	Threonine	−	−	−	−	−	−	−
89	Valine	−	−	−	−	−	−	−
90	Valine/aspartic acid	−	−	−	−	−	−	−
91	2′-Deoxyadenosine	−	−	−	−	−	−	−
92	Inosine	−	−	−	−	−	−	−
93	Thymidine	−	−	−	−	−	−	−
94	Uridine	−	−	−	−	−	−	−
95	Thymidine-*5′*-monophosphate	−	−	−	−	−	−	−
96	Uridine-*5′*-monophosphate	−	−	−	−	−	−	−

a Utilization of β-cyclodextrin, gentiobiose, d-glucosaminic acid, glycerol, maltose, α-methyl-d-galactoside, β-methyl-d-galactoside, isomaltulose, l-rhamnose, turanose, glyoxylic acid, α-hydroxybutyric acid, l-malic acid, asparagine, and phenylalanine was strain dependent (indicated by shading). ddH_2_O, double-distilled water.

b +, oxidized; −, not oxidized; /, critical value.

Liquid chromatography-tandem mass spectrometry (LC-MS/MS) analysis revealed that the major components of the culture supernatant of AM01 to AM06 and ATCC BAA-835 showed no differences under the positive mode (Fig. S2A). However, in the negative mode, the analysis showed that AM06 and ATCC BAA-835 shared the most similarity in the major components of culture supernatant (Fig. S2B). The major components of the culture supernatant of AM02 were similar to those of the culture supernatant of AM03, and the major components of AM04 culture supernatant were similar to those of AM05 culture supernatant (Fig. S2B). However, the major components of AM01 culture supernatant were dissimilar to those of the culture supernatant of any of the above-mentioned strains (Fig. S2B). A comparison of the differential metabolite pathways between AM01 to AM06 and ATCC BAA-835 showed that AM06 and ATCC BAA-835 shared the lowest number of differential metabolite pathways (Fig. S2C). However, the largest number of differential metabolite pathways was found between AM01 and ATCC BAA-835 (Fig. S2C). Analysis of the major fatty acids of AM01 to AM06 and ATCC BAA-835 indicated that 15:0 anteiso fatty acid methyl ester accounted for the highest proportion of the major cellular fatty acids (Table S3).

### Tolerance to artificial gastric and intestinal fluid and bile salts.

To investigate the tolerance of AM01 to AM06 and ATCC BAA-835 to pH changes in the digestive tract, we determined the survival rates of these strains after exposure to artificial gastric and intestinal fluid under different pH conditions and bile salts under different concentrations *in vitro*. AM01 demonstrated the strongest tolerance ability to pH changes, followed by AM06 (Table 3); however, AM06 exhibited stronger tolerance ability to bile salts than any other strain (Table 3). AM02 to AM04 and ATCC BAA-835 could not tolerate acidic and alkaline environments, and their survival rates declined in various degrees after exposure to artificial gastric and intestinal fluid for several hours (Table 3). AM05 could tolerate an alkaline environment but not low pH (Table 3). Bile salts appeared to be suitable for the growth of AM03 to AM06 but not for ATCC BAA-835, AM01, and AM02 (Table 3). Overall, AM06 exhibited the best survival ability to pH changes and different bile salt concentrations.

**TABLE 3:** Strain tolerance to artificial gastric fluid, artificial intestinal fluid, and bile salts

Fluid or salts exposure	Incubation time (h)	% Survival (mean ± SD) of indicated strain						
		ATCC BAA-835	AM01	AM02	AM03	AM04	AM05	AM06
Artificial gastric fluid								
pH 3	1.5	9.77 ± 2.89	84.88 ± 10.07	15.4 ± 12.02	0.18 ± 0.03	34.14 ± 1.76	10.98 ± 1.26	74.97 ± 2.25
	3	2.37 ± 0.58	57.75 ± 8.42	3.71 ± 1.65	0.02 ± 0	9.95 ± 1.38	1.42 ± 0.11	24.4 ± 8.3
pH 2	1.5	14.42 ± 3.79	66.04 ± 18.51	5.63 ± 3.06	31.45 ± 12.09	4.76 ± 1.43	2.43 ± 1.02	15.56 ± 1.39
	3	9.29 ± 3.22	53.20 ± 18.93	2.74 ± 1.16	3.15 ± 1.21	1.87 ± 0.45	1.17 ± 0.32	10.74 ± 1.17
Artificial intestinal fluid								
pH 6.8	4	82.83 ± 1.86	88.13 ± 5.01	79.99 ± 11.89	84.74 ± 7.78	89.35 ± 13.17	129.47 ± 11.61	102.17 ± 12.17
	8	88.75 ± 9.34	107.58 ± 4.83	51.03 ± 10.80	50.435 ± 5.88	124.66 ± 13.14	117.08 ± 9.31	125.27 ± 29.88
Bile salts								
0.5%	4	100.49 ± 10.83	87.96 ± 29.70	101.25 ± 3.20	95.45 ± 8.53	92.73 ± 16.38	123.85 ± 6.98	113.73 ± 12.58
	8	61.83 ± 1.82	70.60 ± 9.90	76.56 ± 2.41	99.47 ± 4.60	144.03 ± 47.64	112.88 ± 7.91	88.47 ± 21.98
1%	4	79.68 ± 5.80	65.02 ± 6.25	84.93 ± 2.49	117.09 ± 9.44	114.96 ± 39.2	114.58 ± 8.52	88.64 ± 7.56
	8	50.57 ± 10.67	49.10 ± 2.83	62.15 ± 2.31	92.83 ± 2.56	106.99 ± 20.92	80.37 ± 12.02	131.31 ± 10.11

### Antibiotic resistance.

To identify the possible drug resistance gene, we used two databases (Antibiotic Resistance Genes Database [ARDB] and Comprehensive Antibiotic Resistance Database [CARD]). There were 13 antibiotic resistance genes referring to 13 types of antibiotics that were annotated in the genomes of AM01, AM04, AM05, and AM06 based on a minimum of 40% amino acid homology with genes in the ARDB, and there were 14 antibiotic resistance genes referring to 20 types of antibiotics that were annotated in the genomes of AM02 and AM03 (Table S4A). Because the ARDB was no longer maintained, we also used the Resistance Gene Identifier (RGI) in the CARD to predict the resistomes of AM01 to AM06. Only one antibiotic resistance gene in the genomes of AM01, AM04, AM05, and AM06 was predicted under strict criteria, and two antibiotic resistance genes were predicted in the genomes of AM02 and AM03 (Table S4B).

Considering the importance of a complete evaluation of the drug resistance of bacterial strains before their clinical application, we selected 16 common types of antibiotics belonging to six types of drug classes base on the prediction of ARDB and CARD for further antibiotic resistance tests. We placed paper disks of different test antibiotics on the agar medium and found that all the strains were resistant to ofloxacin, gentamicin, vancomycin, teicoplanin, norfloxacin, and bacitracin, as predicted (Table 4; Table S4A and B). However, not all strains were resistant to penicillin, cefoperazone, and chloramphenicol, although the resistance genes were annotated (Table 4; Table S4A and B). Moreover, inhibition zones were detected around the paper disks of sulfamethoxazole, tigecycline, and fosfomycin (Table 4), but whether the bacterial strains were sensitive to these antibiotics could not be judged due to inadequate evidence. Importantly, AM01 and AM04 to AM06 exhibited resistance to kanamycin and ciprofloxacin, although they did not harbor any relative antibiotic resistance genes (Table 4; Table S4A and B). As predicted, AM02 and AM03 were resistant to lincosamide, but AM01 and AM04 to AM06 were not, which was confirmed by the drug-sensitive test (Table 4; Table S4A and B). Overall, we found that AM02 and AM03 demonstrated resistance to more types of antibiotics, indicating the need for caution when these two strains are used.

**TABLE 4:** Antibiotic resistance of *A. muciniphila* AM01 to AM06 and ATCC BAA-835 to different antibiotics^*a*^

Drug class	Drug	Inhibition zone diam (mm) (mean ± SD)							Result
		ATCC BAA-835	AM01	AM02	AM03	AM04	AM05	AM06	
Quinolones	Ciprofloxacin	0	0	0	0	0	0	0	Resistance
	Ofloxacin	0	0	0	0	0	0	0	Resistance
	Norfloxacin	0	0	0	0	0	0	0	Resistance
	Sulfamethoxazole	32.50 ± 7.00	36.50 ± 9.00	40.00 ± 6.53	26.50 ± 4.43	34.00 ± 4.90	25.50 ± 3.79	32.00 ± 5.42	IE
Aminoglycosides	Gentamicin	0	0	0	0	0	0	0	Resistance
	Kanamycin	0	0	0	0	0	0	0	Resistance
Tetracyclines	Tigecycline	33.00 ± 5.03	38.00 ± 5.66	34.00 ± 5.16	32.00 ± 4.32	36.00 ± 1.63	31.50 ± 5.26	34.00 ± 4.90	IE
β-Lactams	Penicillin	30.00 ± 4.32	37.50 ± 10.11	38.00 ± 3.27	33.00 ± 4.24	34.50 ± 1.91	26.50 ± 1.00	40.50 ±5.51	Sensitivity
	Cefoperazone	44.50 ± 5.74	37.50 ± 10.12	49.00 ± 10.39	18.00 ± 6.73	47.00 ± 8.25	35.00 ± 14.09	51.00 ±2.58	Sensitivity
Macrolides	Chloramphenicol	44.50 ± 7.55	36.50 ± 10.75	30.50 ± 4.12	32.50 + 3.42	35.50 ± 3.42	28.50 ± 2.52	32.50 ± 3.42	Sensitivity
Others	Vancomycin	0	0	9.50 ± 1.91	9 ± 1.15	0	0	0	Resistance
	Teicoplanin	0	0	0	0	0	0	0	Resistance
	Bacitracin	0	0	0	0	0	0	0	Resistance
	Fosfomycin	35.50 ± 4.43	29.00 ± 1.89	38.50 ± 3.00	32.00 ± 5.16	21.50 ± 3.00	19.50 ± 7.72	30.50 ± 1.91	IE
	Lincosamide	36.50 ± 7.72	36.00 ± 2.31	0	0	43.50 ± 4.12	37.50 ± 3.00	37.50 ± 2.52	Resistance/IE

a IE, evidence and diameter of inhibition were inadequate for determination.

### AM02 and AM06 are genetically and phenotypically stable.

To investigate whether genetic variation occurs during passage, the stability of AM02 and AM06, two strains from different branches of the phylogenetic tree, was evaluated after the strains were incessantly cultured for 100 generations. Single nucleotide polymorphism (SNP) analysis identified two nonsynonymous mutations and one synonymous mutation in the gene region of AM02 B30, B60, and B100 (see “Genetic stability” for an explanation of the designations for strain generations), whereas there was only one nonsynonymous mutation in the gene region of AM02 A30, A60, and A100. For AM06, only one nonsynonymous mutation was detected in the gene region of B60 and B100 (Table S5A). The insertion/deletion (indel) analysis detected three frameshift indels in the gene region of AM02 A60, two frameshift indels in the gene region of A100, but only one non-frameshift indel in the gene region of their parallel controls AM02 B60 and B100. For AM06, one frameshift indel was detected in the gene region of AM06 A30 and B60 and two frameshift indels were detected in the gene region of B100. However, none were detected in their parallel controls (Table S5B). The structural variation (SV) analysis revealed one structural variation in the gene region of AM02 B100, AM06 A100, and B100 (Table S5C). The difference between the parallel controls may be due to the different segments that were captured and analyzed in the indel and SV analyses. In general, the incidence rate of mutation was low during the passage of 100 generations. Therefore, AM02 and AM06 were judged to be genetically stable.

**FIG 2:**
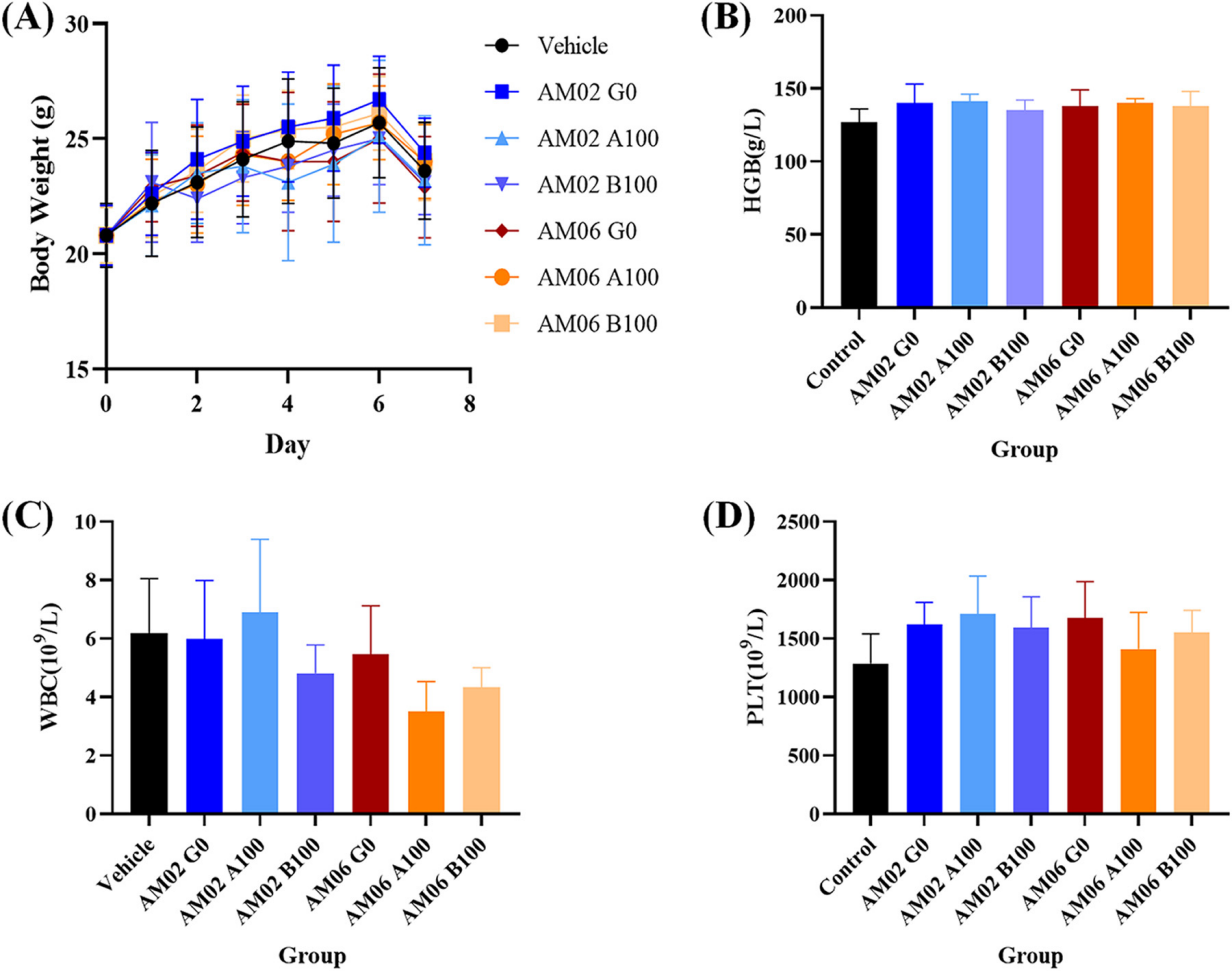
Acute toxicity of the primary and 100th generations of *A. muciniphila* AM02 and AM06 in normal mice. (A) Body weight of mice after 3 days of oral administration of AM02 and AM06. (B) Blood HGB of mice on day 7 after 3 days of oral administration of AM02 and AM06. (C) Blood WBC of mice on day 7 after 3 days of oral administration of AM02 and AM06. (D) Blood PLT of mice on day 7 after 3 days of oral administration of AM02 and AM06.

We also examined phenotypical characteristics and acute toxicity in animals to determine whether the mutations have any effect. Gram staining, scanning electron microscopy, and transmission electron microscopy revealed no differences between the primary and 100th generations of AM02 and AM06. Moreover, mice administered the 100th generation of AM02 and AM06 by gavage for 3 days showed no clinical disorders during the observation period (Fig. 2; Table S6).

### AM02 and AM06 showed no acute toxicity in normal mice.

Normal specific-pathogen-free (SPF) NIH mice were administered the primary and 100th generations of AM02 and AM06 by gavage to confirm the *in vivo* safety of these two strains. No treatment-related death was observed in the NIH mice during the experiments. There was no significant difference in body weight between the groups administered the primary and 100th generations of AM02 and AM06 and saline by gavage, respectively (Fig. 2A). The red blood cell (RBC), hemoglobin (HGB), and platelet (PLT) values showed no significant differences in the routine blood examinations between the groups, indicating that AM02 and AM06 were safe for short-term usage (Fig. 2B to D; Table S6).

### AM06 is nonpathogenic in both normal and immune-deficient mice.

**FIG 3:**
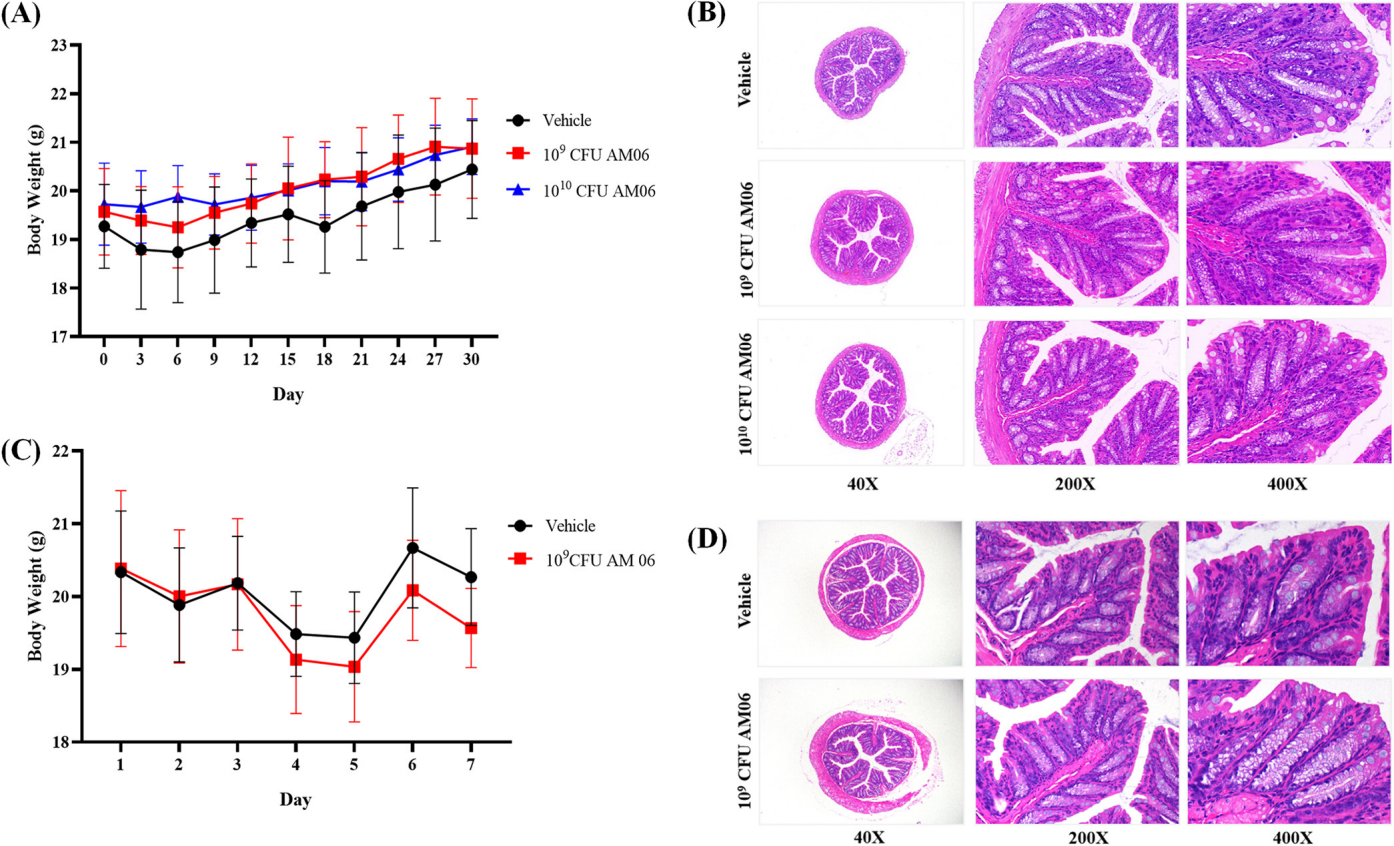
Toxicity of *A. muciniphila* AM06 in normal mice and nude mice. (A) Body weight of BALB/c mice after 1 month of oral administration with 10^9^ and 10^10^ CFU/day of AM06. (B) No injury of colon tissue was observed in the pathology section after 1 month of oral administration of 10^9^ and 10^10^ CFU/day of AM06 to BALB/c mice. (C) Body weight of nude mice after 3 days of oral administration of AM06. (D) No injury of colon tissue of nude mice was observed in the pathological section after 3 days of oral administration of AM06.

In the previous experiment, AM06 showed the best survival ability to pH changes and different bile salt concentrations, resistance to few antibiotics, stable passage for 100 generations, and safe application in NIH mice, which indicate its potential for use as a probiotic strain. To further evaluate the safety of AM06, we administered AM06 to normal SPF BALB/c mice by gavage at low dose (1 × 10^9^ CFU/day) and high dose (1 × 10^10^ CFU/day), respectively, for 1 month. There was no significant difference in body weight between the groups (Fig. 3A), and no obvious histopathological damage was detected in the colon (Fig. 3B). The RBC, HGB, and PLT values showed no significant differences in the routine blood examination between the groups (Table S7). In addition to normal mice, we used immune-deficient nude mice. Still, we observed no death or significant weight loss when these nude mice were orally administered AM06 at a dosage of 1 × 10^9^ CFU/day for 3 days (Fig. 3C). Furthermore, histopathological examination revealed no obvious histopathological damage in the colon of nude mice (Fig. 3D). The routine blood examination showed no significant differences between the groups (Table S8).

## DISCUSSION

Recently, an increasing number of microbial species have been reported to play an important role in modulating the gut microbiota and promoting human health. Studies have shown that several disorders exhibited a strong correlation with gut microbiota (15–17). Consequently, improving human health by modulating the gut microbiota has triggered significant attention in recent years (18). Since its first isolation, *A. muciniphila* rapidly gained public attention because of its beneficial effects on human health, for example, in prevention of obesity, alleviation of ulcerative colitis, and improvement of antitumor efficacy (11, 14, 19). However, some reports show that the beneficial effects of *A. muciniphila* are strain dependent. Studies have shown that different strains of *A. muciniphila* exhibited different effects on colitis and IBD (11, 12). Furthermore, Luna et al. showed that the efficiency of utilizing human milk oligosaccharides by *A. muciniphila* was strain dependent (6). All these findings emphasize the need to consider and evaluate the safety of this bacterium at the strain level before its clinical application. The International Scientific Association for Probiotics and Prebiotics panel also mentioned that robust evidence for the benefits linked to a bacterial strain must be provided before it can be termed a probiotic (20). To date, most studies have focused on the strain *A. muciniphila* ATCC BAA-835. Studies conducted by Derrien et al. based on fluorescence *in situ* hybridization combined with flow cytometry suggested that *A. muciniphila* accounted for 1% to 3% of the total gut microbiota starting from early life (3). Although *A. muciniphila* occupied a certain portion of the total gut microbiota, it was not isolated from feces until 2004 (1). *A. muciniphila* has also been identified by quantitative PCR in human milk samples and was considered to play a crucial role in establishing the primary gut microbiota (5). In the present study, we isolated *A. muciniphila* for the first time from human colostrum. This isolation confirmed the presence of *A. muciniphila* in human breast milk. In addition to the breast milk-derived strain, we isolated other strains from healthy human feces and evaluated their safety before clinical application.

Most studies have focused on improving disorders using *A. muciniphila*, while only a few have discussed its safety. To date, only *A. muciniphila* ATCC BAA-835 and DSM 22959 have been evaluated, and their safety in animals was also confirmed (19, 21). By conducting 16S rRNA V6 amplicon pyrosequencing, Dubourg et al. found that *A. muciniphila*, which accounts for up to 80% of gut microbes, has no impact on health (22). A randomized controlled trial (ClinicalTrials registration no. NCT02637115) applied 10^9^ and 10^10^ CFU/day of live or pasteurized *A. muciniphila* ATCC BAA-835 to individuals with excess body weight and reported that no adverse events or significant changes in relevant safety clinical parameters were observed for 2-week and then 3-month treatments (19, 23). However, the European Food Safety Authority (EFSA) panel did not support this claim of *A. muciniphila* safety because of the limited number of subjects, disease scope, and relevant safety clinical parameters and the low dose of the tested bacterial preparation and short test period (24). No relevant adverse events were observed in the 90-day study, which supported the safety of pasteurized *A. muciniphila* ATCC BAA-835 for use (25). *A. muciniphila* strains other than the ones mentioned in these reports have not been evaluated systematically. According to the FAO/WHO recommendation, we conducted phenotypic testing, analysis of biochemical activity, metabolite production, and major fatty acids, and *in vivo* toxicity testing, combined with genetic analysis, taxonomic identification, and putative virulence and antibiotic resistance gene identification analysis, to evaluate the potential of the strains isolated in our study for use as probiotics (26).

Virulence factor is the primary concern when *A. muciniphila* is being qualified for the probiotic definition. Together, 22 putative virulence factor homologs of AM01, AM04, AM05, and AM06 and 23 putative virulence factor homologs of AM02 and AM03 were annotated in the present study (see Table S2 in the supplemental material). None of these putative virulence factors of *A. muciniphila* had been reported to play a role in any disease. Some of the putative virulence genes are known to play roles in forming lipopolysaccharides and are harbored by most Gram-negative bacteria without causing any endotoxemia, as reported to date (27). The potential pathogenicity is its adherence, related to the initial pathogenic behavior, and degradation of mucosal layers (28). However, *A. muciniphila* is more likely to be a participant in the self-renewal balance of mucosal layers. Unlike other pathogens that invade the inner mucosal layer, *A. muciniphila* primarily colonizes the outer layer (27). The adherence of *A. muciniphila* to the mucus layer is considered a favorable factor for a potential probiotic. *A. muciniphila* participates in maintaining the integrity of the mucosal layer and the thickness of the mucus layer (29). The decreased abundance of *A. muciniphila* may lead to the invasion of pathogens (29).

The toxicity experiment conducted in animals directly indicated the impact of the strain on the body. Studies on germfree and knockout mice reported the safety of applying *A. muciniphila in vivo* (9, 30). Even in tumor-bearing mice, *A. muciniphila* led to a favorable improvement in combination with antitumor therapy (14). To date, no evidence has indicated that the separate application of *A. muciniphila* would convey pathogenic characteristics. However, a study demonstrated that the presence of *A. muciniphila* in Salmonella enterica serovar Typhimurium-infected SIHUMI mice increased the production of proinflammatory cytokines and exacerbated intestinal inflammation (31). This disturbance in host mucus homeostasis may be due to the defective mucus barriers possessed by the germfree mice. Whether a commensal strain would turn into an opportunistic bacterium in susceptible individuals remains a question. In this context, we orally administered *A. muciniphila* AM06 to nude mice and found no abnormal change in the routine blood examination and no death or weight loss in the acute toxicity examination, which provided a rational assumption that a short-term application of AM06 to immunodeficient individuals is safe. We then administered by gavage the primary and 100th generations of AM02 and AM06 to NIH mice for 3 days and observed them for the following 4 days to evaluate acute toxicity. No significant strain-related toxigenic symptoms were detected. All the above-mentioned results suggest that these *A. muciniphila* strains are genetically stable and nonpathogenic after passage.

Antibiotic resistance is another noteworthy issue in the evaluation of probiotic candidates. The concept of the qualified presumption of safety introduced by the EFSA mentioned that the antibiotic resistance genes and the horizontal transfer capabilities of biological agents should be completely evaluated (32). To date, no transferable antibiotic resistance genes of *A. muciniphila* have been reported. In our study, all the antibiotic resistance genes of these *A. muciniphila* strains were present on the chromosome. The guidelines for the assessment of bacterial susceptibility to antimicrobials proposed by the ESFA panel suggested that, at the very least, the antibiotic resistance to ampicillin, vancomycin, gentamicin, kanamycin, streptomycin, erythromycin, clindamycin, tetracycline, and chloramphenicol should be evaluated (33). In the present study, we selected 16 common types of antibiotics belonging to six types of drug classes with reference to the prediction of ARDB and CARD for a complete evaluation of antibiotic resistance. However, we found that the prediction by the drug resistance database was not identical to the *in vitro* experimental result. For instance, in contrast to the prediction, all strains were sensitive to chloramphenicol. Importantly, although aminoglycosides (gentamicin and kanamycin) and teicoplanin did not reach the criteria to be significantly annotated, all the *A. muciniphila* strains in our study showed resistance to them. In addition, it has been reported that *A. muciniphila* is resistant to vancomycin (1). In our study, AM02 and AM03 were not completely inhibited by vancomycin, although they were considered to be “resistant.” In particular, AM02 and AM03 were resistant to lincosamide, but AM01 and AM04 to AM06 were not, which may be attributed to the drug resistance gene existing only in AM02 and AM03. These results emphasize that resistance to antibiotics was strain dependent. In contrast, merely believing in the prediction of the antibiotic resistance database may lead to wrong judgments. Notably, our study results were not completely the same as those reported by Cozzolino et al. in 2020 (21). In that earlier study, *A. muciniphila* DSM 22959, a strain with >99% identity to *A. muciniphila* ATCC BAA-835 in the ANI BLAST search, exhibited resistance to chloramphenicol, clindamycin, streptomycin, and erythromycin and was susceptible to ampicillin, tetracycline, gentamicin, and kanamycin (21). However, in our study, all the strains exhibited resistance to gentamicin and kanamycin and to lincosamide to some extent, which inhibited the growth of *A. muciniphila* ATCC BAA-835, AM01, and AM04 to AM06. In addition to the strain difference, the different culture medium and methods may also lead to different results. The issue of drug resistance becomes more complicated when considering the colonization of commensal bacteria in the intestine. A study reported two cases of increased *A. muciniphila* abundance after the utilization of broad-spectrum antibiotics, including doxycycline, hydroxychloroquine, piperacillin-tazobactam, teicoplanin, and imipenem (22). However, in the same study, *A. muciniphila* ATCC BAA-835 was sensitive to imipenem (MIC = 0.7 mg/L), doxycycline (MIC = 0.38 mg/L), and piperacillin-tazobactam (MIC = 0.7 mg/L) *in vitro* (22). Under the pressure of broad-spectrum antibiotics, *A. muciniphila* may acquire drug resistance genes from other colonized bacteria (34). Therefore, probiotic candidates should be thoroughly screened and evaluated before and after their clinical application.

### Conclusion.

**FIG 4:**
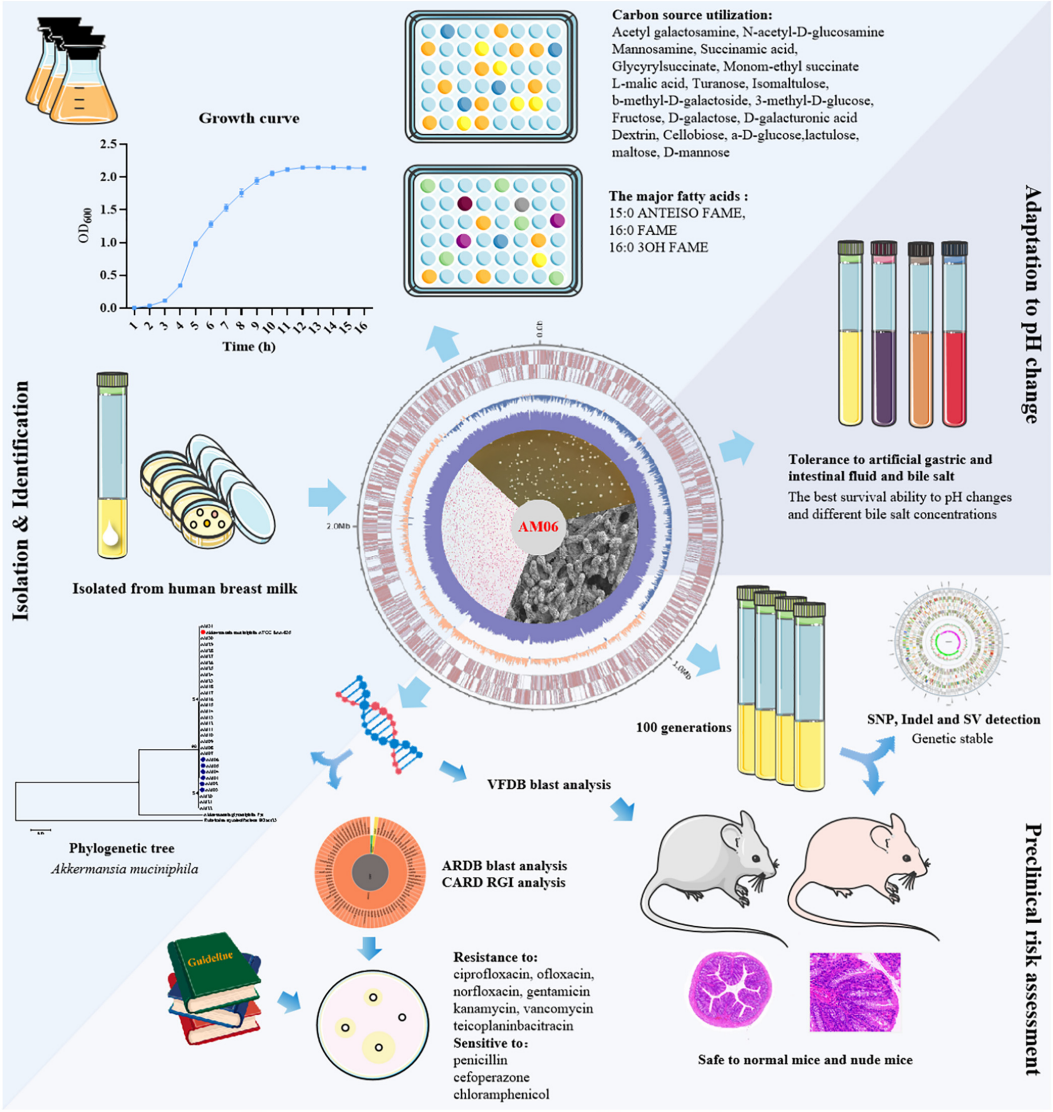
Isolation and evaluation of general characteristics, genes, stability, resistance to antibiotics, tolerance to artificial gastrointestinal fluids, and toxicity at the animal level suggest that AM06 is a safe *A. muciniphila* strain for use as a probiotic candidate.

To summarize, we evaluated multiple *A. muciniphila* strains in terms of different aspects, including the origin, general characteristics, genes, stability, resistance to antibiotics, tolerance to artificial gastrointestinal fluids, and toxicity at the animal level. Overall, AM06, the *A. muciniphila* strain isolated from human breast milk, was confirmed to have no virulence factors (Fig. 4). Its tolerance to pH changes and bile salts indicates its desirable probiotic properties. AM06 is genetically stable and nonpathogenic to both normal mice and nude mice, which further ensures its safety as a probiotic candidate. Considering these findings together, we propose that AM06 is a safe *A. muciniphila* strain and exhibits great potential for use as a probiotic candidate.

## MATERIALS AND METHODS

### Bacterial strains, culture conditions, and identification.

Fecal samples from five healthy volunteers and breast milk from one lactating woman were collected. Briefly, 5 g of fecal sample or 0.5 mL of breast milk sample was mixed with 50 mL of Ringer’s solution to obtain a suspension that was diluted according to doubling dilution in mucin liquid medium (1). The doubling dilution sample was then streak-inoculated on basal mucin agar medium and BHI agar medium containing 0.1 g/L *N*-acetylglucosamine and 0.315 g/L hemin to obtain colonies at 37°C under anaerobic conditions for 2 to 5 days. All colonies were first identified using *A. muciniphila*-specific primers F (5′-CAGCACGTGAAGGTGGGGAC-3′′) and R (5′-CCTTGCGGTTGGCTTCAGAT-3′′) (9). Next, matrix-assisted laser desorption ionization–time of flight mass spectrometry (MALDI-TOF MS) using the Autof ms1000 system and 16S rRNA gene sequencing were performed to confirm the possible colonies (35). A total of 31 *A. muciniphila* strains (designated AM01 to AM31) were isolated in this experiment. Akkermansia muciniphila ATCC BAA-835 was purchased from the American Type Culture Collection (ATCC).

For a translational perspective, a culture medium (unpublished data) containing no animal-derived components was used to culture all the *A. muciniphila* strains in the following experiments.

### Genome sequencing, assembly, and analysis.

A DNA extraction kit (Qiagen, USA) was used to extract the total DNA from all the bacterial strains. PCR amplification was conducted on the strains using universal primers (27F, 5′-AGAGTTTGATCCTGGCTCAG-3′′; 1492R, 5′-GGTTACCTTGTTACGACTT-3′′) to obtain 16S rRNA genes. The 16S rRNA gene sequences of all colonies were compared with the sequences obtained from GenBank by BLAST analysis. Pairwise sequence alignment was conducted using the program MEGA 7 on all possible colonies that were identified as *A. muciniphila* with an identity of >98.7% by BLAST analysis. We then aligned the cloned sequences and 16S rRNA gene sequences of the nearest relatives and generated a phylogenetic tree using the neighbor-joining method through MEGA 7.

The complete genome sequence of all *A. muciniphila* strains was sequenced using the Illumina NovaSeq PE150 system (Novogene Co., Ltd.). Raw short-read sequences were filtered before the data were assembled using SOAP denovo (version 2.04), SPAdes, ABySS, and CISA software. Gene prediction was performed using GeneMarkS (version 4.17) software. Putative virulence factors were annotated by BLAST analysis with genes in the Virulence Factors of Pathogenic Bacteria Database (VFDB) based on a minimum of 50% amino acid homology (36). The putative antibiotic resistance genes were annotated by BLAST analysis with genes in the ARDB and by using the RGI with genes in the CARD (37, 38). The ANI was calculated by BLAST analysis of strains AM01 to AM06 and strain ATCC BAA-835.

### General characteristics.

The bacterial strains were cultured anaerobically for 24 to 36 h before Gram staining. Their microscopic morphology characterization was determined by scanning electrochemical microscopy. To evaluate motility, the bacteria were cultured anaerobically on a semisolid medium (solidified with 0.5% agar) at 37°C for 3 to 5 days. For generating growth curves, the bacterial strains were cultured anaerobically in 1% (vol/vol) liquid medium at 37°C for 42 h. The OD_600_ value was recorded every 2 to 5 h.

Carbon source utilization analyses were conducted using a Biolog AN MicroPlate test panel (Biolog, USA) (39). The bacterial cells were cultured in advance on culture agar medium containing no animal-derived components. The colonies of strains AM01 to AM06 and ATCC BAA-835 were evenly inoculated into AN-IF buffer (Biolog, USA) to obtain a suspension that was inoculated into the test plate. After anaerobic incubation for 20 to 24 h, the test plates were analyzed using a microplate reader (SpectraMax M2). Deionized water was used as a negative control. Supernatant samples of late-logarithmic-phase AM01 to AM06 and ATCC BAA-835 cultures were collected by centrifugation at 15,000 × *g* for 5 min and then filtered using a 0.22-μm filter before analysis by LC-MS/MS.

The major fatty acids were detected using an Agilent 6890 gas chromatograph and analyzed using the Sherlock microbial identification system (MIDI) (40).

### Antibiotic resistance testing.

Colonies of AM01 to AM06 were inoculated into sterile phosphate-buffered saline (PBS) to obtain a suspension. The suspension was adjusted to an OD_600_ value of 1. Next, 100 μL of the adjusted suspension was distributed onto the BHI agar medium containing 0.1 g/L *N*-acetylglucosamine and 0.315 g/L hemin. Paper disks of different test antibiotics were placed on the agar medium. After anaerobic cultivation of the culture medium for 3 to 5 days at 37°C, the diameter of inhibition zones was measured, and judgment was made based on EUCAST expert rules on antimicrobial susceptibility testing (41). Test antibiotics were selected based on the antibiotic resistance genes of AM01 to AM06 annotated by BLAST analysis with genes in the ARDB based on a minimum of 40% amino acid homology and by RGI with genes in the CARD under strict criteria.

Paper disks of the test antibiotics were purchased from Beijing Tiantan Biological Products Co., Ltd. The specifications for each antibiotic paper disk are as follows: ciprofloxacin, 5 μg/disk; ofloxacin, 5 μg/disk; norfloxacin, 10 μg/disk; sulfamethoxazole, 23.75/1.25 μg/disk; gentamicin, 120 g/disk; kanamycin, 30 μg/disk; tigecycline, 15 μg/disk; penicillin, 10 IU/disk; cefoperazone, 75 μg/disk; chloramphenicol, 30 μg/disk; vancomycin, 30 μg/disk; teicoplanin, 30 μg/disk; bacitracin, 0.04 IU/disk; penicillin, 10 IU/disk; fosfomycin, 200 μg/disk; and lincosamide, 2,200 μg/disk.

### Tolerance to artificial gastric and intestinal fluid and bile salts.

Live probiotics must tolerate highly acidic gastric fluid, alkaline intestinal fluid, and bile salts before colonizing suitable sites and functioning in the digestive tract. Therefore, we exposed AM01 to AM06 and ATCC BAA-835 to artificial gastric and intestinal fluid and bile salts to observe whether they can survive in an acidic or alkaline environment. The pH of human gastric fluid fluctuates between 2.5 and 3, and in extreme situations, the lowest pH can reach 1.5 and the highest pH can be >6, depending on the food intake (42). In general, food passes through the gastrointestinal tract in less than 4 h. To simulate gastric fluid, we prepared a sterile HCl solution (pH 2 and 3) with 10 g/L pepsin. Each bacterial strain was cultured in the HCl solution for 1.5 to 3 h. The numbers of CFU of each strain in different pH solutions at 1.5 and 3 h were enumerated. The pH of the intestinal fluid is stable at approximately 6.8. We exposed the bacterial strains to artificial intestinal fluid (pH 6.8) and determined their CFU count at 2 and 4 h. Because bile salts in the intestine can destroy the cell membrane and alter cell permeability, resulting in cell death, we also exposed the bacterial strains to a solution of bile salts (Oxoid) at 0.5% and 1% (vol/vol) and determined the CFU count of each strain at 4 and 8 h to evaluate their tolerance to bile salts.

### Genetic stability.

We subcultured AM02 and AM06 repeatedly for 100 generations to explore whether genetic variation occurs during passage. Parallel controls A and B were designated for each strain. Subsequent passages were performed by inoculating 1% of each culture into fresh medium every 24 h, beginning with primary generation (designated G0) and until the 100th generation was obtained. SNP, indel, and SV analyses of the primary, 30th, 60th, and 100th generations of each parallel control of the strains (designated G0, A30, B30, A60, B60, A100, and B100 of AM02 or AM06) were conducted to evaluate whether genetic variation occurred. SNP refers primarily to the DNA sequence polymorphism caused by the single nucleotide variation at the genome level, including transition and transversion. Indel refers to the insertion and deletion of small fragments in the genome. SAMTOOLS was used to detect the individual SNP and the insertion and deletion of small fragments (<50 bp), as well as to analyze the variation of SNP/indel in the functional regions of the genome (43). The parameters of SAMTOOLS were as follows: mpileup -m 2 -F 0.002 -d 10000 -u -L10000. SV refers to the insertion, deletion, inversion, and translocation of large segments at the genome level. The insertion, deletion, inversion, intrachromosomal translocation (ITX), and interchromosomal translocation (CTX) between the reference and the sample were determined using BreakDancer software (v1.4.4) (44) with the parameters -q 20-d prefix.

Morphological variations were evaluated by Gram staining, scanning electron microscopy, and transmission electron microscopy of the primary and 100th generations of AM02 and AM06. Acute toxicity of the 100th generation of AM02 and AM06 was evaluated in SPF mice, as described below.

### Toxicity to normal mice and nude mice.

To evaluate acute oral toxicity, 6- to 8-week-old female SPF NIH mice were procured from Boji Medical Technology Co., Ltd. A total of 42 NIH mice were randomly assigned to seven groups (*n* = 6). Each group was administered by gavage a bacterial suspension containing 1 × 10^9^ CFU/day of the primary or 100th generation of culture supernatant of AM02 and AM06 for 3 days, which were designated AM02 G0, AM02 A100, AM02 B100, AM06 G0, AM06 A100, and AM06 B100, respectively. A100 and B100 were the parallel controls of the 100th generation of the bacterial culture. Saline was administered at 0.5 mL/day as the control. The general condition of the mice was monitored, and body weight was measured daily for 7 days. On day 7, blood was sampled for routine examination and biochemical tests were done with the mice under isoflurane anesthesia after fasting for 24 h.

For further safety evaluation of the AM06 strain, 10 female SPF BALB/c mice aged 6 to 8 weeks from each group, which were procured from Beijing Vital River Laboratory Animal Technology Co., Ltd., were administered by gavage an AM06 suspension containing 1 × 10^9^ or 1 × 10^10^ CFU/day, respectively, for 1 month. Saline was administered at 0.5 mL/day as the control. The general condition of the mice was observed daily, and body weight was measured every 3 days. BALB/c nude mice, which were procured from Beijing Vital River Laboratory Animal Technology Co., Ltd., were orally administered AM06 at a dosage of 1 × 10^9^ CFU/day for 3 days, and the general condition and body weight of the mice were observed for 7 days. At the end of the study, the mice were fasted for 24 h, after which blood was sampled for routine examination under isoflurane anesthesia. Then the mice were sacrificed and their organs were sampled for histopathological examination. All animal experiments were performed with the approval of the Animal Ethics Committee of the Beijing Institute of Microbiology and Epidemiology, Beijing, China (agreement no. IACUC-DWZX-2017-006).

### Statistical analysis.

The antibiotic resistance test and tolerance test were performed in triplicate, and values were analyzed using IBM SPSS Statistics 25 and are expressed as means ± standard errors.

In the acute toxicity assay, data were analyzed by Levene’s test to determine whether they were distributed normally and exhibited homogeneity of variance. Analysis of variance was performed when data were distributed normally and exhibited homogeneity of variance, whereas the Kruskal-Wallis test was performed to determine statistically significant differences when data were not distributed normally and exhibited homogeneity of variance. A *P* value of <0.05 was considered to be statistically significant.

### Ethics approval and consent to participate.

This study was approved by the Ethics Committee of Nanfang Hospital (Guangzhou, China; agreement no. NFEC-2019-144). All the study volunteers signed a consent document and consented to the isolation of gut bacteria from their feces or breast milk.

### Data availability.

All data generated or analyzed during this study are included in this published article (and its supplemental material).

The sequence data were summitted to the SRA database under accession number PRJNA915585.
