# Supplementary material for: Safety Evaluation and Probiotic Potency Screening of Akkermansia muciniphila Strains Isolated from Human Feces and Breast Milk
Source: Microbiol Spectr. 2023 Feb 14;11(2):e03361-22. doi: 10.1128/spectrum.03361-22 (PMC10103750; doi:10.1128/spectrum.03361-22)
Supplement: Supplemental file 9 — Fig. S1 and S2. Download spectrum.03361-22-s0001.pdf, PDF file, 2.9 MB [file spectrum.03361-22-s0001.pdf]

Fig. S1

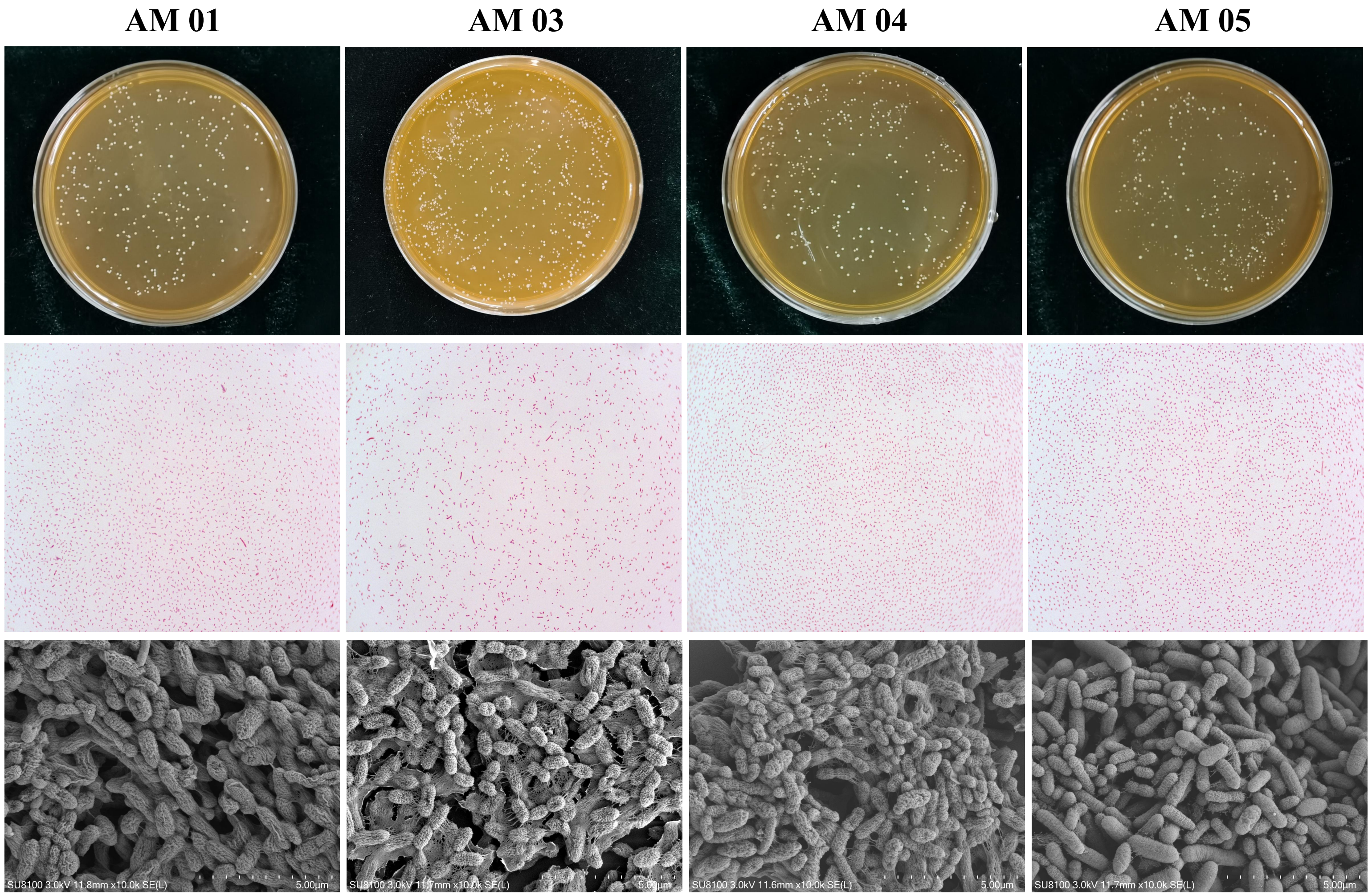
AM 01
AM 03
AM 04
AM 05
SU8100 3.0kV 11.8mm x10.0k SE(L)
5.00μm
SU8100 3.0kV 11.7mm x10.0k SE(L)
5.00μm
SU8100 3.0kV 11.6mm x10.0k SE(L)
5.00μm
SU8100 3.0kV 11.7mm x10.0k SE(L)
5.00μm

Figure S1. Bacterial colonies on agar medium, gram staining under the optical microscope, and morphological characteristics of bacterial cells under the scanning electron microscope of *A. muciniphila* AM01 and AM03–05.

Fig. S2

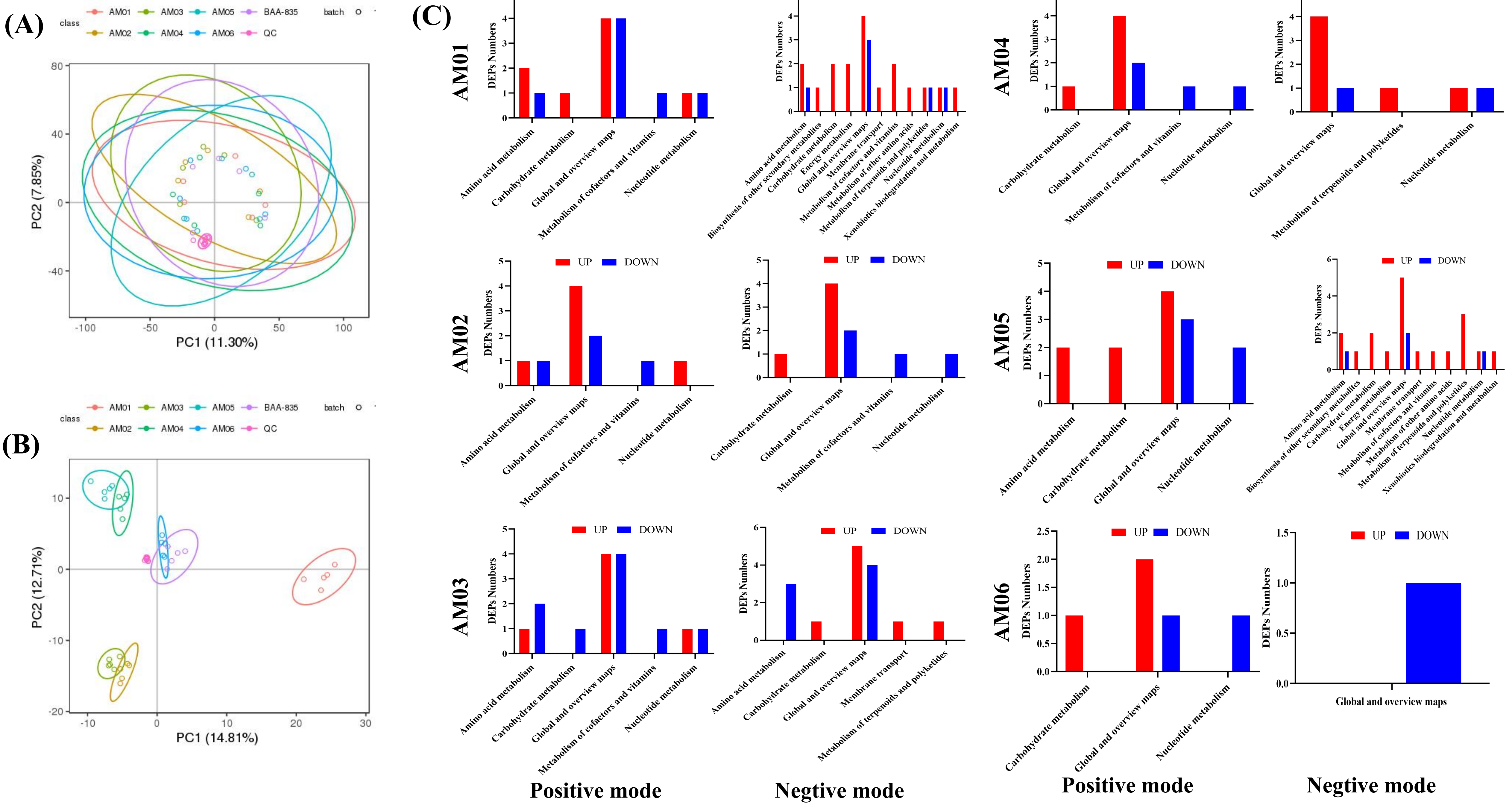
(A)
class
AM01
AM02
AM03
AM04
AM05
AM06
BAA-835
QC
batch
PC1 (11.30%)
PC2 (7.85%)
(B)
PC1 (14.81%)
PC2 (12.71%)
(C)
AM01
AM02
AM03
AM04
AM05
AM06
UP
DOWN
DEPs Numbers
Amino acid metabolism
Carbohydrate metabolism
Global and overview maps
Metabolism of cofactors and vitamins
Nucleotide metabolism
Biosynthesis of other secondary metabolites
Energy metabolism
Membrane transport
Metabolism of other amino acids
Metabolism of terpenoids and polyketides
Xenobiotics biodegradation and metabolism
Positive mode
Negtive mode
Positive mode
Negtive mode

Figure S2. Major components of the culture supernatant of *A. muciniphila* AM01–06 and *A. muciniphila ATCC BAA-835* under positive mode (A) and negative mode (B). (C) Differential metabolite pathways between *A. muciniphila* AM01–06 and *A. muciniphila ATCC BAA-835*.
