# Supplementary material for: Safety Evaluation and Probiotic Potency Screening of Akkermansia muciniphila Strains Isolated from Human Feces and Breast Milk
Source: Microbiol Spectr. 2023 Feb 14;11(2):e03361-22. doi: 10.1128/spectrum.03361-22 (PMC10103750; doi:10.1128/spectrum.03361-22)
Supplement: Supplemental file 10 — Legends of Fig. S1 and S2 and of Tables S1 to S8. Download spectrum.03361-22-s0010.docx, DOCX file, 0.01 MB [file spectrum.03361-22-s0010.docx]

**Supplementary figures and tables ledgends**

**Supplementary Figure 1.** Bacterial colonies on agar medium, gram staining under the optical microscope, and morphological characteristics of bacterial cells under the scanning electron microscope of *A. muciniphila* AM01 and AM03–05.

**Supplementary Figure 2.** Major components of the culture supernatant of *A. muciniphila* AM01–06 and *A. muciniphila* *ATCC BAA-835* under positive mode (A) and negative mode (B). (C) Differential metabolite pathways between *A. muciniphila* AM01–06 and *A. muciniphila* *ATCC BAA-835*.

**Supplementary Table 1.** Sample origin of 31 AKK colonies. AM06, AM04, and AM23-31were isolated from different samples from a same volunteer at different ages.

**Supplementary Table 2.** Putative virulence factors of *A. muciniphila* in the genomes of AM01–06 based on a minimum of 50% amino acid homology with genes in the VFDB.

**Supplementary Table 3.** Major fatty acids of *A. muciniphila* AM01–06.

**Supplementary Table 4.** (A) Putative antibiotic resistance genes identified in the genome of *A. muciniphila* AM01–06 (ARDB). (B) Putative antibiotic resistance genes identified in the genome of *A. muciniphila* AM01–06 (CARD).

**Supplementary Table 5.** Number of SNPs (A), InDels (B), and SV (C) between different generations of *A. muciniphila* AM02 and AM06.

**Supplementary Table 6.** Blood biochemical test results of mice under 3-day oral administration with AM02 and AM06.

**Supplementary Table 7.** Blood routine of mice under 1-month oral administration with AM06.

**Supplementary Table 8.** Blood routine of nude mice under 3-day oral administration with AM06.
